# Study of Carrier Mobilities in 4H-SiC MOSFETS Using Hall Analysis

**DOI:** 10.3390/ma15196736

**Published:** 2022-09-28

**Authors:** Suman Das, Yongju Zheng, Ayayi Ahyi, Marcelo A. Kuroda, Sarit Dhar

**Affiliations:** 1 Department of Physics, Auburn University, Auburn, AL 36849, USA; 2 SemiQ Inc., Lake Forest, CA 92630, USA

**Keywords:** 4H-SiC MOSFET, nitridation, scattering, Hall measurements, body bias, transverse electric field

## Abstract

The channel conduction in 4H-SiC metal–oxide–semiconductor field effect transistors (MOSFETs) are highly impacted by charge trapping and scattering at the interface. Even though nitridation reduces the interface trap density, scattering still plays a crucial role in increasing the channel resistance in these transistors. In this work, the dominant scattering mechanisms are distinguished for inversion layer electrons and holes using temperature and body-bias-dependent Hall measurements on nitrided lateral 4H-SiC MOSFETs. The effect of the transverse electric field ($E_{eff}$) on carrier mobility is analyzed under strong inversion condition where surface roughness scattering becomes prevalent. Power law dependencies of the electron and hole Hall mobility for surface roughness scattering are determined to be $E_{eff}^{-1.8}$ and $E_{eff}^{-2.4}$, respectively, analogous to those of silicon MOSFETs. Moreover, for n-channel MOSFETs, the effect of phonon scattering is observed at zero body bias, whereas in p-channel MOSFETs, it is observed only under negative body biases. Along with the identification of regimes governed by different scattering mechanisms, these results highlight the importance of the selection of substrate doping and of $E_{eff}$ in controlling the value of channel mobility in 4H-SiC MOSFETs.

## 1. Introduction

Silicon carbide (4H-SiC) is one of the primary wide-band-gap semiconductors for high power and harsh environment applications because of its physical properties, such as a high critical electric field and high thermal conductivity [1]. Discrete 4H-SiC diodes and metal–oxide–semiconductor field effect transistors (MOSFETs) are being widely adopted for high voltage power conversion in hybrid/electric vehicles [2], solar and wind energy generation [3], and various high temperature applications [4], enabling significant advances for next-generation energy efficient power systems. Moreover, 4H-SiC is a unique candidate for the development of an integrated circuit (IC) technology that is still in its infancy. 4H-SiC IC technology operating at very high temperatures >300 °C [5,6] is very attractive, as it enables operation in environments and ambientes that are not accessible to conventional silicon or silicon on insulator (SOI) platforms [7,8]. Lateral complementary metal–oxide–semiconductor (CMOS) IC technology in 4H-SiC is desirable for the fabrication of large-scale integration devices due to its high noise immunity and low static power consumption [9]. To be materialized, such technology demands both n- and p-channel MOSFETs capable of operating at high-temperatures. While n-channel MOSFETs have reached a suitable degree of maturity through the nitridation of the SiO_2_/4H-SiC interface [10,11,12], mechanisms governing electrical transport in their p-channel counterparts must be investigated to reach comparable levels of development in terms of channel conductivity and device stability.

Recent studies [10,13] on 4H-SiC MOSFETs use field-effect mobility models to analyze channel transport. Additionally, research by Mikami et al. [13] explored the importance of body bias and explained how a body bias experiment can provide predictions of transistor characteristics for MOSFETs made with higher or lower substrate doping using field effect mobility on the weak inversion region. However, the channel scattering mechanisms for p-channel 4H-SiC MOSFET remain unexplored using Hall analysis. Hall mobility is more accurate than field effect mobility, as the carrier concentration is measured independently and does not get affected by trapped charges. A comprehensive study for both kinds of carriers will enable control of the operation region and the preferable substrate doping concentrations for high-temperature IC design. To this end, the channel mobility of nitric oxide (NO)-annealed n- and p-channel 4H-SiC MOSFETs in the strong inversion regime was characterized using Hall measurements. Measurements were performed using the body-bias technique, which allows variation of the effective electric field ($E_{eff}$) in the channel [13,14]. This approach allows one to differentiate and establish the dominant scattering mechanisms limiting carrier transport, in different carrier density and substrate doping regimes. The power law that governs the relation between $\mu_{Hall}$ and $E_{eff}$ was determined for both electrons and holes in the surface-roughness-scattering-dominant regime. In addition, it is demonstrated that a combination of temperature and body bias enables observation of the dominant mechanisms transitioning from surface roughness to phonon scattering.

## 2. Materials and Methods

### 2.1. Fundamentals on Channel-Transverse Electric Fields and Mobility

The channel’s resistance depends on the channel’s inversion-carrier concentration and its mobility ($\mu_{Hall}$), which are related to the atomic-scale composition, structure, and defects at the SiO_2_/4H-SiC interface. The mobile carrier’s concentration is reduced by traps at electrically-active defect sites, and the channel’s mobility is degraded by the interfacial scattering processes. When the MOSFFET is on, the dominant scattering mechanisms at a SiO_2_/4H-SiC interface that limits $\mu_{Hall}$ are: (i) Coulomb scattering occurring due to trapped charges present at or near the interface oxide and from ionized impurities in the depletion region close to the channel; (ii) phonon scattering occurring due to the interaction of the carriers with lattice vibration in the channel and the surface; and (iii) surface roughness due to imperfections of the 4H-SiC interface [15,16,17]. The resulting carrier mobility is given by Matthiesen’s rule [18]:

(1) $$\frac{1}{\mu_{Hall}}=\frac{1}{\mu_{C}\;}+\frac{1}{\mu_{ph}}+\frac{1}{\mu_{SR}}\;$$

Here, $\mu_{C}$, $\mu_{ph}$, and $\mu_{SR}$ are the Coulomb, phonon, and surface roughness scattering limited mobilities, respectively. 

On Si MOSFETs [19,20], earlier studies suggest that the transverse electric field $E_{eff}$ is an important parameter for influencing scattering in the channel. The magnitude of $E_{eff}$ can be obtained as [19],

(2) $$E_{eff}=\frac{1}{\epsilon }\left(\sqrt{2N_{A}q\epsilon .\left(2\phi_{B}\pm V_{BS}\right)}+\eta qn_{s}\right)\;$$

**Figure 1:**
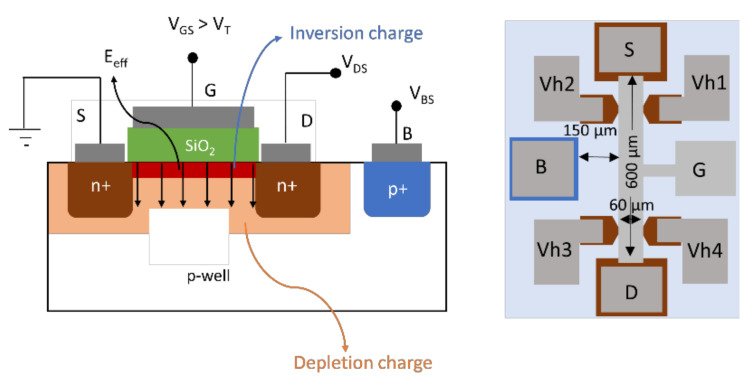
(**left**) Schematic diagram of lateral n-channel Hall MOSFET with the transverse electric field $E_{eff}$ shown, which depends on space charge and inversion charge in the channel at a gate voltage higher than the threshold voltage. (**right**) Top view of a schematic of the fabricated Hall bar MOSFET. Vh1, Vh2, Vh3, and Vh4 represent the Hall voltage terminals.

The first term in Equation (2) represents the amount of depletion charge, and the second term stands for the induced inversion layer charge excluding trapped charges. Here the “+” sign is applicable for n-channel MOSFETs and the “−“ sign is for p-channel MOSFETs. A schematic diagram of these two charge distributions is shown in the left panel of Figure 1. In Equation (2), η is a constant with a value of 0.5 for n-channel and 0.33 for p-channel Si MOSFETs at $27{\;}^{{}^{\circ}}C$ [19,20,21,22], which are considered the same for 4H-SiC [13,14,23,24], but this choice does not affect the results and may need further studies in a wider effective field range. ϵ is the dielectric constant, *q* is the electron charge, $n_{s}$ is the free carrier concentration, and *N_A_* is the p-well substrate doping concentration for n-channel MOSFETs. *V_BS_* is the substrate or body to source bias. $\phi_{B}=\frac{KT}{q}\;\ln\frac{N_{A}}{n_{i}}$ is the bulk potential. K is the Boltzmann constant and $n_{i}$ denotes the temperature-dependent intrinsic carrier concentration. Note, for p-channel MOSFET, the n-well substrate doping concentration is denoted by *N_D_*. Application of body/substrate bias acts as a separate knob to control the depletion width without affecting the channel carrier concentration. A variation of channel electric field to study its effect on the conduction and the effects of substrate doping concentration is possible through *V_BS_*.

### 2.2. Experimental Methods

Hall bar MOSFETs with a channel length of 600 μm and a channel width of 60 μm were fabricated on a 4° off-axis (0001), Si-face oriented 4H-SiC substrate with a p-type epilayer (*N_A_* − *N_D_* = 6 × 10^15^ cm^−3^) for n-channel MOSFETs and an n-type epilayer (*N_D_* − *N_A_* = 6.2 × 10^15^ cm^−3^) for p-channel MOSFETs. Additional to the Hall bar MOSFETs, T-gated MOSFETs were also present in the chip with a 200 μm × 200 μm channel (width, length). The cross-sectional schematic of the Hall bar device is shown in Figure 1 (left). A nominally uniform doping profile was created using nitrogen and aluminum ion implantations at 700 °C to form n+ and p+ layers on the MOSFETs, respectively, to form source, drain and body contacts. The implanted layers were then activated by annealing at 1650 °C for 30 min in flowing Ar with a graphitic cap to protect the surface. After removal of the cap, gate oxidation was carried out at 1150 °C for 10 h followed by post oxidation annealing at 1175 °C for 2 h in NO. The thickness of the gate oxide was measured to be about ~60 nm and 55 nm by the capacitance voltage method (CV) for n- and p-channel MOSFETs, respectively. Contact photolithography and reactive ion etching were carried out to remove the oxide from the source/drain/body (S/D/B) regions and Hall voltage (V_h_) terminals. For n-channel MOSFET, Al was evaporated for all the contacts and annealed at 800 °C for 30 min in flowing Ar. On the other hand, for p-channel MOSFET, Ti was sputtered and lifted off to define the S/D/B/V_h_ contacts, as shown in Figure 1 (right), following which annealing at 1000 °C for 2 min was performed in flowing Ar to form ohmic contacts. Subsequently, aluminum (55 nm) was thermally evaporated as the gate metal and patterned using lift-off. An overlayer of Au/Cr was sputtered on all the contacts of both kinds of MOSFETs. Finally, the Hall bar MOSFET was mounted on a ceramic chip with epoxy and wire bonded to gold pads on the chip. 

## 3. Results and Discussions

### 3.1. Classification of the Dominant Scattering Mechanisms

**Figure 2:**
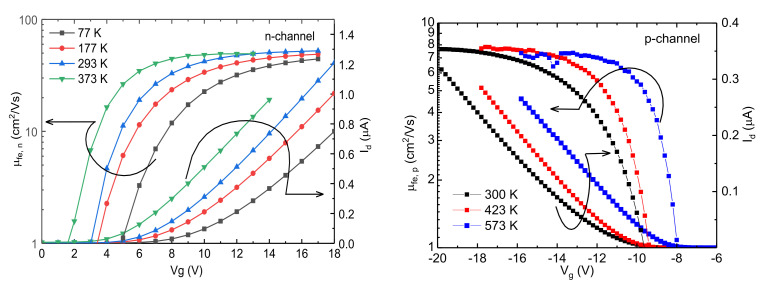
Field effect mobility of electrons and holes versus gate voltage at different temperatures for n- and p-channel 4H-SiC MOSFETs. For n-channel MOSFETs, channel width/length was 60 µm/600 µm, oxide thickness was 60 nm, V_ds_ was set to 0.4 V, and temperature was varied from 77 to 373 K. For p-channel MOSFETs, channel width/length was 200 µm/200 µm, oxide thickness was 55 nm, V_ds_ was set to 0.1 V, and temperature was varied from 300 to 573 K.

After fabrication of the 4H-SiC MOSFETs, transfer characteristics were obtained at various temperatures. The temperature for the n-channel MOSFETs was varied from 77 to 373 K. For the p-channel MOSFETs, on the other hand, the temperature was raised above room temperature (300 K) until 573 K. The absolute value of threshold voltage became smaller at higher temperatures, as shown in Figure 2. This is associated with the reduction of occupied interface trap densities (D_it_) at higher temperatures [25]. In addition, the field effect mobility for electrons ($\mu_{fe,\;n}$) was observed to increase from 77 to 296 K. For the p-channel MOSFET, the field effect mobility of holes was observed to be weakly dependent on temperature under strong inversion. 

**Figure 3:**
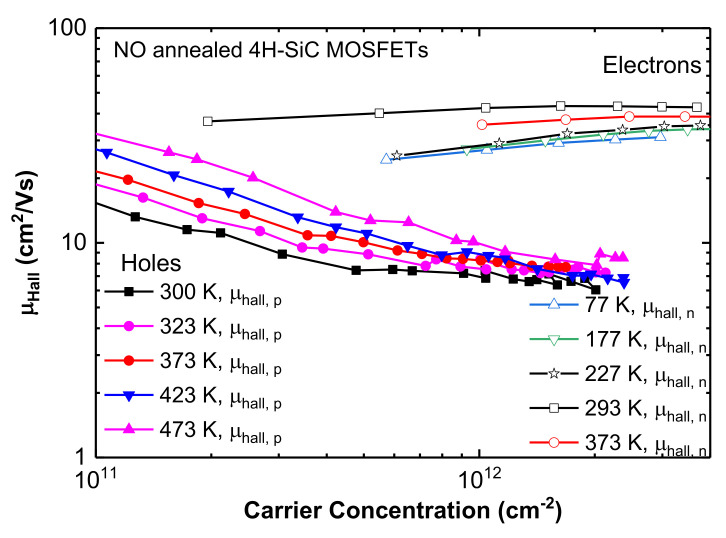
Typical Hall mobility as a function of carrier concentration at different temperatures for n- and p-channel MOSFETs with channel width 60 µm and channel length 600 µm for both kinds of Hall bar MOSFET, and V_ds_ was set to 0.75 V.

To confirm the dominant scattering mechanisms in these devices, Hall measurements were carried out under a perpendicular magnetic field of 0.6 T. At a high gate voltage overdrive, the Hall and field effect mobilities merge, and at low and intermediate gate voltages, field effect mobilities are lower than Hall mobilities due to the presence of trapped charges (see supplementary material). Figure 3 shows $\mu_{Hall}$ versus carrier concentration curves at different temperatures and at zero body bias for electrons and holes in n- and p-channel 4H-SiC MOSFETs. To obtain $\mu_{Hall}$ versus carrier concentration plot, the gate to source voltage $V_{g}$ was varied from +2 to +10 V for n-channel and −6 to −20 V for p-channel MOSFETs at constant drain to source voltage $V_{DS}=0.75\;V$. The threshold voltage was found to be +5 V for electrons (at 293 K) and −10 V for holes (300 K), based on linear extrapolation of the I_D_–V_G_ characteristics (Figure 2). For n-channel MOSFETs, Hall mobility above room temperature drops. An increase in mobility from 77 to 293 K is a signature of prevalent Coulomb scattering, whereas above 293 K, mobility decreases with increasing temperature when phonon scattering is dominant. Therefore, for electrons, transport is limited by Coulomb and phonon scattering. From the trend of the experimental results in figure in Section 3.3 and other published results [23,26], electron mobility will reduce below 77 K due to increased Coulomb scattering. Above 373 K, mobility would also decrease, but due to increased phonon scattering. The presence of surface roughness scattering at higher concentration can also be observed in earlier reports [14]. Conversely, for holes in p-channel MOSFET, Hall mobility is seen to increase at lower carrier concentration (for approximately $p_{s}<6\times 10^{11}\;{\mathrm{cm}}^{-2}$ at 300 K), signifying dominant Coulomb scattering, and becomes independent of temperature at approximately $p_{s}=10^{12}\;{\mathrm{cm}}^{-2}$ (see supplementary materials). Temperature-independent mobility is a sign of surface roughness scattering. Therefore, hole mobility in 4H-SiC p-channel MOSFET is limited by Coulomb scattering at weak inversion and dominant surface roughness scattering at strong inversion.

After distinguishing the dominant scattering mechanisms in these devices, Hall measurements were carried out under the effect of body bias (*V_BS_*) so that the Hall mobility ($\mu_{Hall}$) can be studied as a function of transverse electric field $\left(E_{eff}\right)$ in the strong inversion regime. Recent studies [13,14] observed the effect of transverse electric field on mobility in the strong inversion regime maintaining a fixed carrier concentration. A constant carrier concentration realizes fixed screening from scattering centers and enables the assessment of the sole effect of surface roughness scattering on mobility. 

### 3.2. Analysis of Surface Roughness Scattering Using Body Bias Measurements

**Figure 4:**
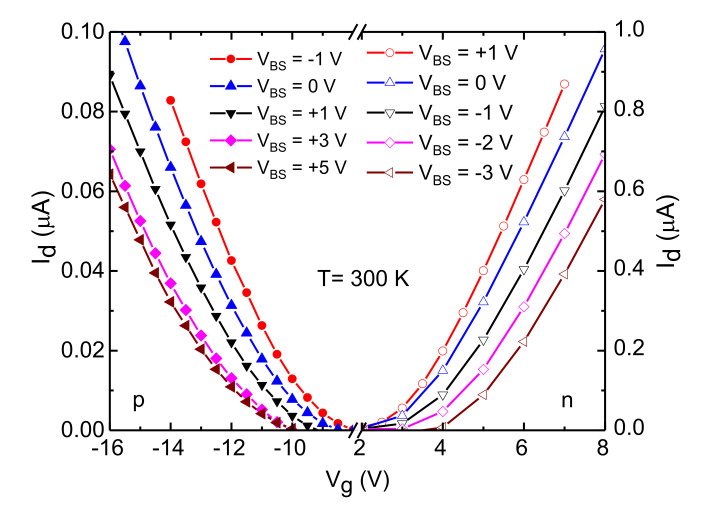
Transfer characteristics at room temperature (300 K) for different body biases (V_BS_) for n- and p-channel MOSFETs. I_d_–V_g_ sweeps were taken at a constant drain-to-source voltage (0.75 V). A change in V_th_ can be observed due to the modulation of the depletion layer width at the body to source junction with the application of body bias. Note that the y-axis scales are different for the two kinds of MOSFETs.

First, to confirm the body bias effect on each transistor, a transfer characteristic at different body bias levels was measured, as shown in Figure 4. The threshold voltages (*V_th_*) at 300 K and *V_BS_* = 0 were approximately +4 V and −10 V for n- and p-channel MOSFETs respectively, based on linear extrapolation of the I_D_–V_G_ characteristics. It can be observed that the threshold voltage increases as the *V_BS_* goes from forward to reverse bias for both types of MOSFETs. 

**Figure 5:**
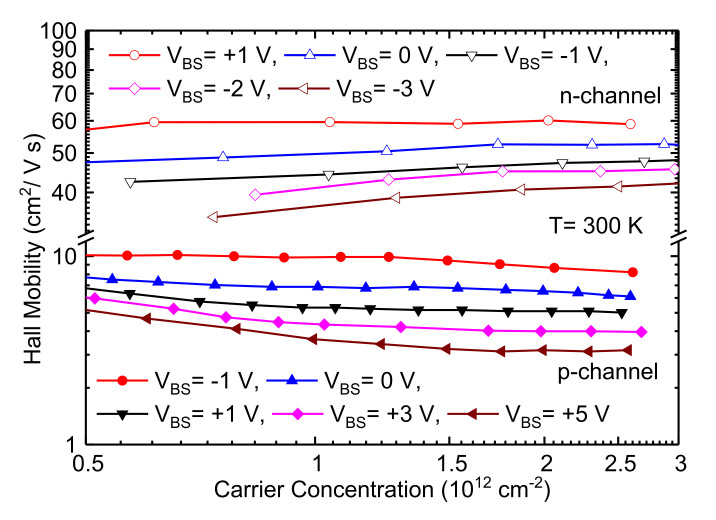
Hall mobility as a function of channel carrier concentration at different body biases at 27 °C. The body bias was varied from +1 to −3 V for n-channel MOSFETs and −1 to +5 V for p-channel MOSFETs; the gate bias V_GS_ was swept to modulate the carrier concentrations under a fixed body bias. A forward body bias value decreases the depletion layer width between source and body, increasing mobility, and at reverse body bias, *E_eff_* increases due to expansion of depletion layer, which in turn decreases the channel mobility.

A positive increment in the *V_BS_* widens the depletion layer between the source and the body junction, thereby requiring a higher gate voltage to achieve same level of drain current (*I_d_*) as in lower *V_BS_*. The plot of $\mu_{Hall}$ as a function of carrier concentration in the strong inversion region for different body biases is shown in Figure 5. An increase in reverse body bias increases the magnitude of the transverse electric field (Equation (2)) and pushes the carriers closer to the surface, making the channel width thinner (~1 nm) at strong inversion. 

Hence, at a high gate voltage, when mobile holes are closer to the surface, surface roughness scattering becomes even more prevalent through the influence of the varying perturbed potential energy [15]. This is attributed to charge carrier wave functions becoming more susceptible to the fluctuating perturbation near the interface stemming from the crystal miscut (4° off axis substrate) and nano-steps/roughness. As a result, an increased $E_{eff}$ lowers the value of $\mu_{Hall}$. Conversely, a negative body bias decreases the value of $E_{eff}$, which widens the channel region and lessens the impact of the surface roughness scattering. 

**Figure 6:**
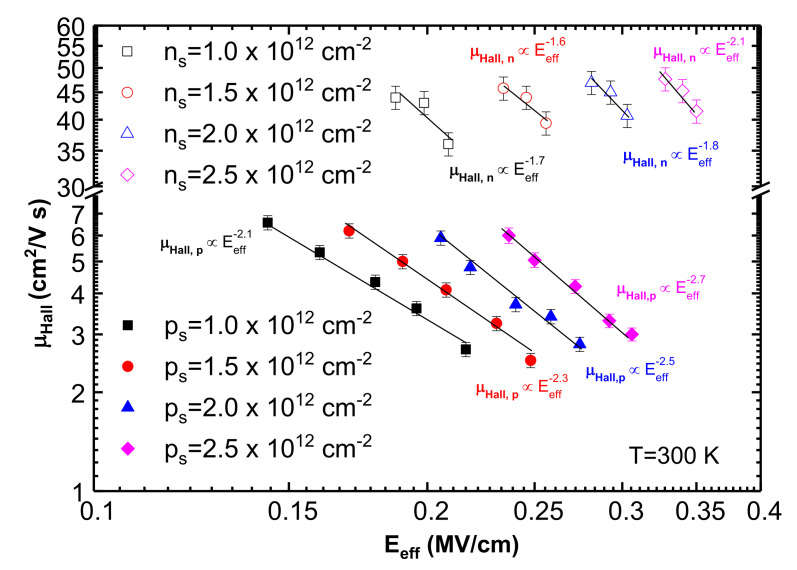
Transverse electric field (*E_eff_*) dependence of Hall mobility ($\mu_{Hall}$) at different body biases (−1, −2, and −3 V for n-channel and 0, +1, +3, +5, and +7 V for p-channel) at room temperature (27 °C) for different carrier concentrations ($1.0\times 10^{12},1.5\times 10^{12},\;2.0\times 10^{12},\;\mathrm{and}\;2.5\times 10^{12}$ cm^−2^), in log–log scale. The different *E_eff_* points for a particular curve were obtained from different body biases at a fixed carrier concentration from Figure 5. The average power law that $\mu_{\mathrm{Hall}}$ follows on $E_{eff}$ is $\left(-1.8\pm 0.2\right)$ for n-channel MOSFETs and $\left(-2.4\pm 0.3\right)$ for p-channel MOSFETs, providing the power of $E_{eff}$ for surface roughness scattering for electrons and holes in 4H-SiC MOSFETs. Error bars signify ~5% error estimated based on variation in at least 3 measurements.

These results were used to extract the functional dependence of $\mu_{Hall}$ at a fixed carrier concentration to analyze surface roughness scattering under constant screening. For this purpose, using Equation (2), $n_{s}\;\mathrm{and}\;p_{s}$ were converted to $E_{eff}$ for each *V_BS_* above 0 V in the strong inversion regime. Next, $\mu_{Hall}$ was extracted at fixed carrier concentrations ($1.0\times 10^{12},1.5\times 10^{12},\;2.0\times 10^{12}\;\mathrm{and}\;2.5\times 10^{12}$ cm^−2^) and plotted against $E_{eff}$, as shown in Figure 6. The values of *V_BS_* were chosen above zero volts, where the surface roughness becomes the dominant mechanism and there is a minimal contribution from phonon scattering. The curves have been fit using a power law function of $E_{eff}$, yielding exponents of $\left(-1.8\pm 0.2\right)$ and $\left(-2.4\pm 0.3\right)$ for channel electrons and holes in 4H-SiC n- and p-channel MOSFETs, respectively. These values are close to those found earlier for surface roughness scattering in Si MOSFETs [20], suggesting that at high $E_{eff}$, surface imperfections have a similar effect on channel carriers in 4H-SiC as in Si MOSFETs. However, differently from Si, where $\mu_{Hall}$ versus $E_{eff}$ curves merge with each other when surface roughness scattering becomes the dominant regime, this universal behavior is not visible for 4H-SiC in Figure 6. This is consistent with earlier studies on 4H-SiC n-channel MOSFETs [14,23,24,27,28]. Perhaps for a single 4H-SiC MOSFET, the measurable range of $E_{eff}$ is not large enough before the breakdown of the gate dielectric to observe the merging of $\mu_{Hall}$ at higher $E_{eff}$, and a larger range of measurements using devices with different substrate doping is necessary, as reported in [24]. 

### 3.3. Phonon-Scattering-Limited Mobility

**Figure 7:**
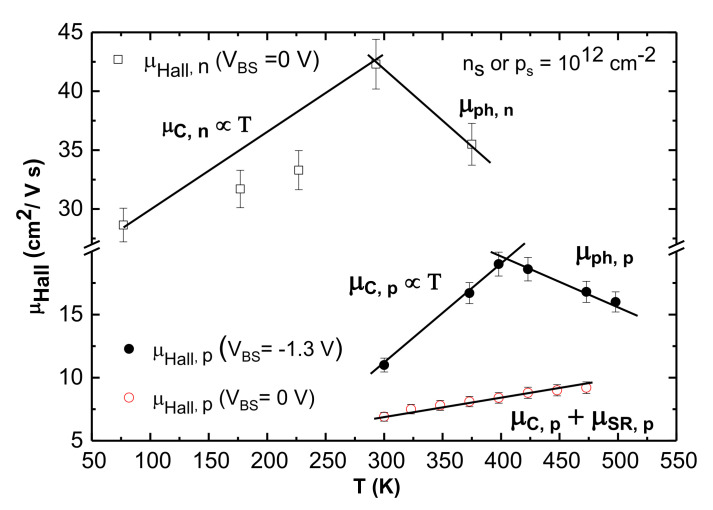
Hall mobility ($\mu_{Hall}$) versus temperature T for different body biases is plotted for a fixed $n_{s}\;/{\mathrm{or}\;p}_{s}=10^{12}$ cm^−2^. For n-channel MOSFET, Coulomb scattering is dominant below RT; above RT phonon scattering can be observed at zero body bias. However, for p-channel MOSFETs, $\mu_{\mathrm{Hall}}$ is limited by a combined effect of Coulomb and surface roughness scattering, *V_BS_* = 0 V. For *V_BS_* = −1.3 V, $\mu_{Hall}$ increases linearly until 398 K (125 °C) due to Coulomb scattering. At higher temperatures, phonon scattering dominates and $\mu_{\mathrm{Hall}}$ decreases with temperature as T^−0.9^. Error bars signify variation in in at least 3 measurements.

For n-channel MOSFETs, dropping mobility was observed above room temperature at zero body bias, which signifies the dominance of phonon-scattering-limited mobility, as seen in Figure 7. This fact is consistent with recent work [23] which reported that phonon-scattering-limited mobility can be observed for n-channel MOSFETs fabricated on lightly doped ($≲5\times 10^{15}$ cm^−3^) p-type epitaxial layers. However, for p-channel MOSFETs, phonon-scattering-limited mobility was absent at zero body bias in the measured temperature range. In this case, phonon scattering was visible only when the source to body junction was kept at a forward bias (*V_BS_* < 0). Then, the power law-dependence of the $\mu_{Hall}$ on $E_{eff}$ was observed to give an exponent of −0.32 (see supplementary materials). A power law of $E_{eff}^{-1/3}$ is an indication of phonon scattering [20,23]. In our study, a negative body bias was used to replicate a lightly doped n-type substrate through the “effective doping concentration” [14] of the n-well $N_{D,eff}$, given by:

(3) $$N_{D,eff}=N_{D}\left(1+\frac{V_{BS}}{2\phi_{B}}\right).$$

At a body bias $V_{BS}=-1.3\;V$ and a fixed $p_{s}=10^{12}$ cm^−2^, a low value of $N_{D,eff}$ (approximately $3\times 10^{15}$ cm^−3^) can be maintained in the 300 to 498 K (27 °C to 225 °C) temperature range. Figure 7 shows that $\mu_{Hall}$ increases from room temperature to 398 K due to Coulomb scattering, after which it decreases owing to the dominance of phonon scattering following a power law of $T^{-0.9}$.

The power of T found here is near to the theoretical value of T^−1^ [29]. Under negative $V_{BS}$, the channel is thick enough to interact with the n-well lattice vibrations at *T* > 398 K, and the hole mobility is limited by phonon scattering. This is consistent with n-channel 4H-SiC MOSFETs fabricated on lightly doped substrates, where phonon-scattering-limited mobility at low $E_{eff}$ or $N_{D,eff}$ can be observed [23]. Conversely, for $V_{BS}=0$ V, channel thickness is comparatively thinner than at $V_{BS}=-1.3\;V$. In this case, no photon-limited scattering is observable, but $\mu_{Hall}$ is limited by the cumulative effect of Coulomb and surface roughness scattering. As a result, $\mu_{Hall}$ increases slightly at higher temperatures. 

## 4. Conclusions

In conclusion, the dominant scattering mechanisms in n- and p- 4H-SiC MOSFET channels were distinguished using Hall measurements. Electron mobility in n-channel MOSFET is limited by Coulomb scattering in weak inversion and a combination of phonon and surface roughness scattering in the strong inversion region. On the other hand, hole mobility in p-channel MOSFETs is limited primarily by Coulomb and surface roughness scattering. Body-bias- and temperature-dependent Hall measurements were performed and analyzed under strong inversion to study mobility in terms of effective transvers electric field ($E_{eff}$). As body bias is increased, a higher value of $E_{eff}$ confines the mobile carriers near the 4H-SiC/SiO_2_ interface, resulting in surface-roughness-scattering-limited mobility. A changing body bias was applied to determine the power-law dependence of channel electron and hole mobility on the $E_{eff}$ for surface roughness scattering in 4H-SiC as $E_{eff}^{-1.8}$ and $E_{eff}^{-2.4}$, respectively. Furthermore, with the application of negative *V_BS_*, the depletion layer between the source and body contact decreases, and the resulting low $E_{eff}$ leads to higher mobility at a given gate bias. For p-channel MOSFET, at a forward *V_BS_* (−1.3 V at 398 K), the channel is thick enough to interact with the n-well lattice vibration, giving rise to a phonon-scattering-limited hole mobility. Therefore, at low negative *V_BS_*, the channel conduction behaves like a MOSFET with a lightly doped substrate. These new findings emphasize the significance of substrate doping selection for 4H-SiC MOSFETs. Additionally, for the development of highly efficient 4H-SiC CMOS devices for high temperature operation, the three types of scattering processes identified here must be considered to depend on the value of the transverse electric field in the channel and the operating temperature.

## Data Availability

The data that support the findings of this study are available from the corresponding authors upon reasonable request.

## Supplementary Materials

The following supporting information can be downloaded at https://www.mdpi.com/article/10.3390/ma15196736/s1. Figure S1: Field effect versus Hall mobility. Figure S2: Surface roughness scattering at a high hole concentration. Figure S3: Hole mobility versus transverse electric field for different body biases at room temperature.
