# Supplementary material for: Study of Carrier Mobilities in 4H-SiC MOSFETS Using Hall Analysis
Source: Materials (Basel). 2022 Sep 28;15(19):6736. doi: 10.3390/ma15196736 (PMC9571812; doi:10.3390/ma15196736)
Supplement: Supplementary file 1 [file materials-15-06736-s001.zip › materials-1922783-supplementary.pdf]

Supplementary Materials

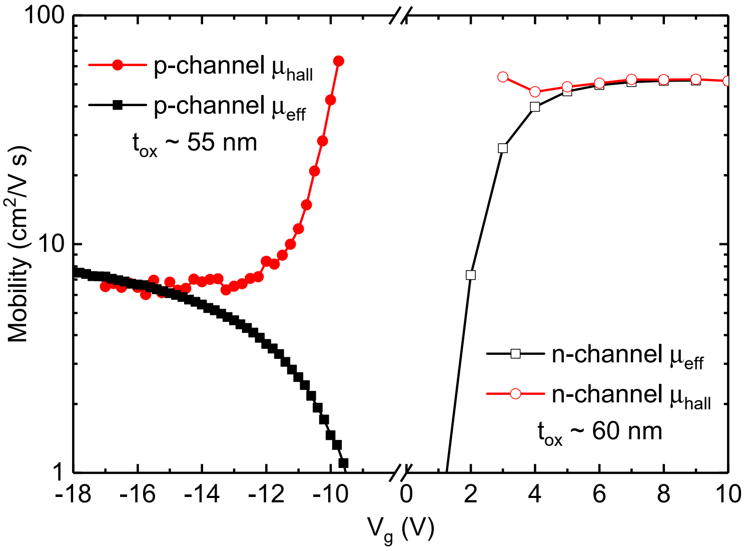

Figure S1: Field effect and Hall mobility for nitrided n- and p-channel MOSFETs at room temperature and zero body bias as a function of gate voltage. The oxide thicknesses are 55 and 60 nm for n- and p-channel MOSFETs respectively.

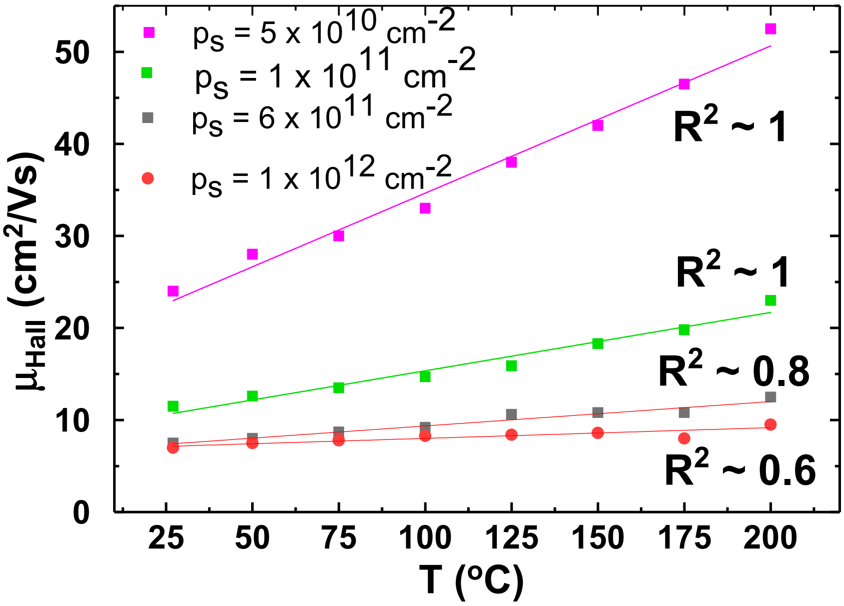

Figure S2: Temperature dependence of the Hall mobility for p-channel MOSFET at different carrier concentration. At p_s = 1x12 cm^{-2}, the fitting parameter R^2 can be seen to deviate from 1, showing surface roughness scattering regime.

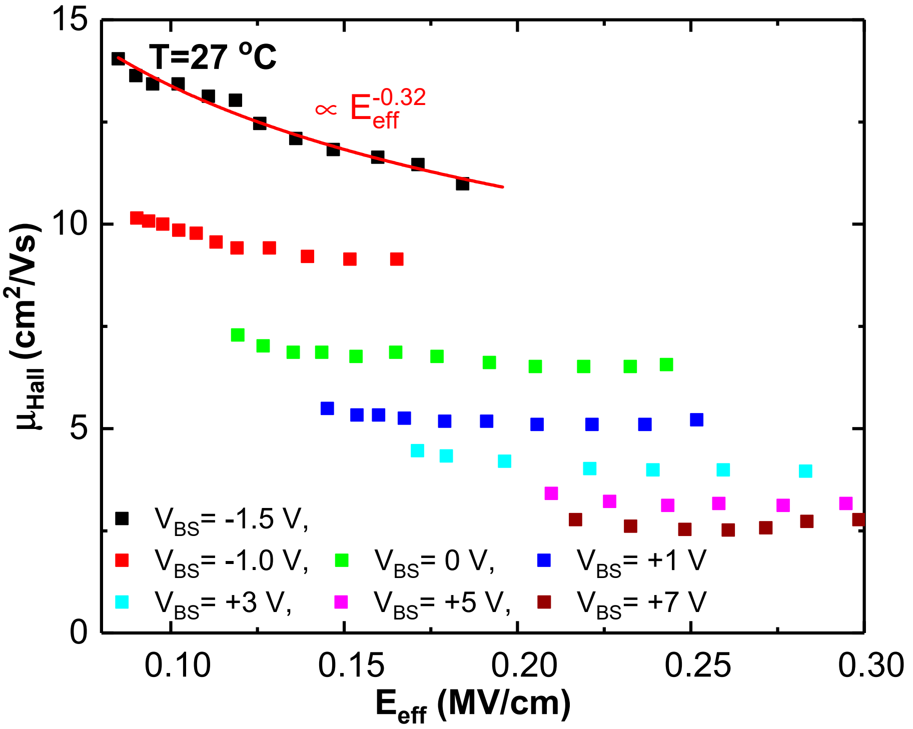

Figure S3: Hole mobility versus transverse electric field for different body biases at room temperature. The curve for V_{BS} =-1.5 V shows a dependence of $E_{eff}^{-1/3}$ which is an indication of phonon scattering.
